# A CASE TO ILLUSTRATE THE ROLE OF OPHTHALMOLOGIST IN SYSTEMIC LUPUS ERYTHEMATOSUS

**DOI:** 10.4103/0019-5154.70686

**Published:** 2010

**Authors:** Vasudev Anand Rao, Datta Gulnar Pandian, Nirupama Kasturi, V Muthukrishanan, D M Thappa

**Affiliations:** *From the Department of Ophthalmology, Jawaharlal Institute of Postgraduate Medical Education and Research (JIPMER), Pondicherry - 605 006, India*; 1 *From the Department of Dermatology and STD, Jawaharlal Institute of Postgraduate Medical Education and Research (JIPMER), Pondicherry - 605 006, India*

**Keywords:** *Systemic lupus erythematosus*, *macular infarction*, *India*, *lupus retinopathy*, *unilateral*

## Abstract

Systemic lupus erythematosus (SLE) affects the eye as part of the disease or due to the drugs used in therapy. Ocular involvement is seen in one third of the patients with SLE. SLE is rare in India and found less frequently in males and children. SLE retinopathy is usually bilateral. We report an unusual case of unilateral macular infarction in a boy caused by systemic lupus erythematosus. A fourteen year old boy was presented with skin rashes and loss of vision in left eye. Posterior segment examination showed hyperemic edematous disc, arteriolar attenuation, venous dilatation, multiple cotton wool spots around the disc and macula in the left eye. There was no improvement in vision with pulse steroids and cyclophosphamide. The clinical implication of SLE retinopathy is that the disease is severe and warrants systemic immunosuppressive therapy. SLE-induced macular infarction is rare and has poor visual prognosis. As serious ocular complications of SLE can be silent, routine ophthalmological evaluation is warranted in all patients.

## Introduction

Systemic lupus erythematosus (SLE) is a chronic autoimmune multisystem disease wherein the eye is involved as a part of active lupus or antiphospholipid antibody syndrome or due to drugs used in the treatment of SLE. SLE is rare in India with a prevalence of 3 in 100, 000.[1] The median age of onset is 24.5 years and the sex ratio (F:M) is 11:1.[1] Majority of patients (90%) are female.[1]. Ocular manifestations affecting the eye or the visual system are seen in 25 – 33% of patients with SLE.[2,3] Ocular involvement may precede or follow the systemic illness. SLE retinopathy is a common ocular manifestation secondary to keratoconjuctivitis sicca.[2,4] About 10-20% of patients of SLE have a childhood onset of the disease.[5] SLE-induced macular infarction is uncommon and is usually bilateral. We report a case of sight threatening unilateral SLE retinopathy in a boy.

## Case Report

**Figure 1:**
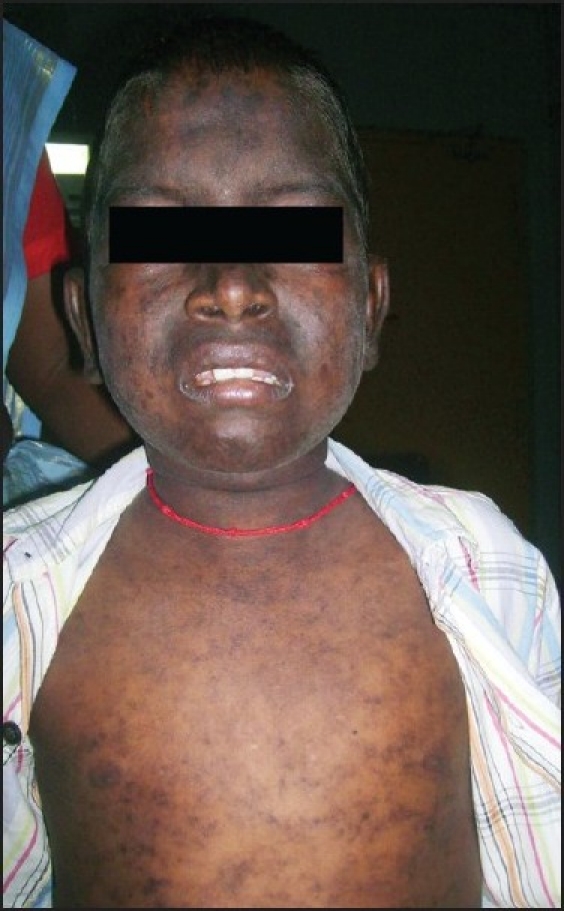
Discoid rashes on face and trunk

A fourteen-year-old boy presented with diminished vision in the left eye, fever, and headache for two weeks. He had hair loss over the occiput, discoid rashes over the face, forehead, trunk and both lower limbs that exacerbated in sun light [Figure 1]. He was a recently diagnosed case of SLE treated with hydroxychloroquine 20 mg/day. His visual acuity was 20/40 in the right eye and was hand movements close to face with accurate projection of rays in the left eye. Anterior segment examination was normal in both eyes and there was no relative afferent pupillary defect. Posterior segment examination showed hyperemic edematous disc, arteriolar attenuation, venous dilatation, multiple cotton wool spots around the, and macula with macular edema in the left eye. Right eye had no evidence of SLE retinopathy. In view of poor vision with extensive cotton-wool spots at the macula, absence of relative afferent pupillary defect, macular infarction of left eye was diagnosed.

The investigation revealed that his erythrocyte sedimentation rate was elevated, 80 mm/hr. LE cells were positive in serum. Anti ds DNA and ANA tests were positive. His leucocyte count was within normal limits. Skin biopsy showed hyperkeratotic papillomatosis, follicular plugging, and dermis showed mild focal periadnexal inflammatory infiltrate. Renal parameters were within normal limits. Magnetic resonance imaging of brain was within normal limits.

**Figure 2:**
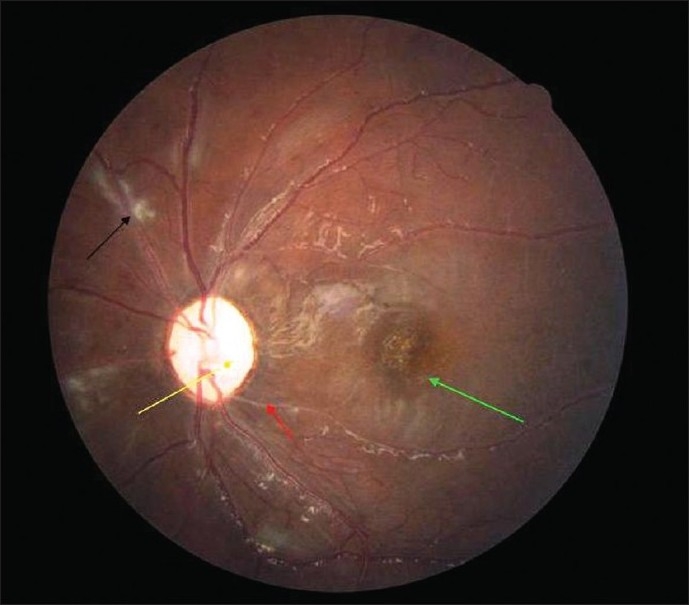
Left eye fundus. Yellow arrow – Disc pallor (optic atrophy), Red arrow – vascular sheathing, Green arrow – Macular scarring, Black arrow – Cotton wool spot

Later he developed irrelevant speech, forgetfulness, alteration in behavior, hence CNS lupus was suspected. He also had palpable purpura, which disappeared in 2 days. He was started on antibiotics and pulse methyl prednisolone followed by a course of oral steroids. Single pulse cyclophosphamide provided symptomatic relief and reduced neuropsychiatric symptoms. This did not improve the vision in the left eye. There was no effect in the right eye in 4 months follow up. The left eye fundus findings, on follow up, were optic atrophy, macular scarring, sheathing of vessels and cotton wool spots [Figure 2]. The macular edema and cotton wool spots at macula regressed with residual macular scarring and optic atrophy.

## Discussion

Cutaneous manifestations are seen in 85% of cases, which includes malar rash, discoid rash with photosensitivity and alopecia.[6] Other manifestations include arthritis, glomerulonephritis, serositis, hematological, and central nervous system (CNS) disturbances.[2,6] Ocular manifestations are seen in one third of the cases with a form of discoid lupus erythematosus of the eyelids or episcleritis, scleritis or secondary Sjögren’s syndrome. It can also be sight threatening in the form of lupus retinopathy.[4]

The various pathogenic mechanisms implicated in SLE are immune complex deposition, vasculitis and thrombosis.[4] Histopathologicaly infiltration of vessel wall with fibrillar material causing vascular constriction and hyaline thrombus formation is seen[2,7] This manifests as cotton wool spots, retinal vein or artery occlusion and macular infarction.[3] It causes perivascular lymphocyte infiltration and the vessel wall is free of inflammatory cells, hence not causing a true vasculitis.[2] Antiphospholipid/antineuronal antibody-mediated cytotoxicity results in retinal cell death and optic nerve demyelination.[4]

Retinal affection occurs in 7-26%[3,4] of SLE patients and are invariably bilateral. Lupus retinopathy may be asymptomatic or may result in permanent visual impairment. SLE-induced microvasculopathy is characterized by cotton wool spots, retinal hemorrhages, perivascular exudates and vascular tortousity. In patients positive for antiphospholipid antibody, vaso-occlusive conditions such as CRAO, CRVO, and BRVO are common.[7,8] Choroidal involvement can manifest as subretinal fluid accumulation, retinal pigment epithelium detachments or central serous chorioretinopathy.[4] Toxic maculopathy can result from hydroxychloroquine therapy but only at a dose of >6.5 mg/kg/d for more than 5 years.[4] Ocular involvement in SLE is usually silent unless its complications cause blindness. Complications such as vitreous hemorrhage, tractional retinal detachment, secondary neovascular glaucoma, secondary to proliferative retinopathy and optic atrophy are rare but sight threatening.[4,7] Routine ophthalmic referral can aid in early diagnosis and treatment with laser photocoagulation and vitrectomy, preventing visual loss in proliferative lupus retinopathy.[7]

Macular infarction can present as the initial manifestation of SLE.[9,10] It causes marked loss of vision and rarely responds to high dose systemic corticosteroids or other immunosuppressive therapy and causes poor visual prognosis.[10] Macular infarction is not found to be correlated with elevated antiphospholipid antibody levels.

Scleritis and SLE retinopathy are hallmark of systemic disease activity and requires systemic immunosuppresion.[4] Treatment for SLE includes photoprotection, hydroxychloroquine, systemic steroids, immunosuppressive agents such as cyclosporine, azathioprine, chlorambucil, and plasmapheresis. Lupus retinopathy has been shown to correlate well with raised antiphospholipid antibody levels.[3] It has been shown that patients with SLE retinopathy are at a higher risk of developing CNS lupus (71%) when compared to those without retinopathy (13%).[2]

## Conclusion

The manifestations of SLE are diverse. Eye involvement may be sight threatening or an indicator of systemic disease activity and reflects systemic vascular damage. The serious ocular manifestation such as lupus retinopathy usually requires systemic immunosuppressant and antithrombotic agents in order to reduce the ocular morbidity associated with the disease. Hence, ophthalmic evaluation is mandatory for patients with SLE.
